# The British Pharmaceutical Codex

**Published:** 1907-10-19

**Authors:** 


### The Hospital

A Journal of

The Medical Sciences and Hospital Administration.

New Series. No. 30, Vol. II. [No. 1102, Vol. XLIII.] SATURDAY, OCTOBER 19, 1907.

### THE BRITISH PHARMACEUTICAL CODEX.

The publication of the British Pharmaceutical Codex, under the authority of the Pharmaceutical Society of Great Britain, is an event which must necessarily be of considerable interest to the medical profession. For though the endorsement of the Pharmaceutical Society has no legal force or value, it will certainly be recognised as carrying a large moral influence, and thus pharmacists generally may be expected to turn to the volume a ready and obedient ear. The main purpose of the Codex is to provide an accepted and uniform definition of drugs and medicinal preparations as these are prescribed throughout the British Empire. At present there exists towards this end the British Pharmacopœia, as issued under the authority of Parliament by the General Medical Council. It is, however, practically impossible that an official publication can appear at frequent intervals, nor is it altogether desirable that such a volume shall be subject in successive issues to considerable or revolutionary changes. The aim is rather to preserve a steady standard, and to make only such changes as circumstances render necessary. Further, whether wisely or unwisely, custom seems to demand that no drug shall be admitted into the pages of the national Pharmacopœia until time and the event have afforded a guarantee of its therapeutic value. For these and other reasons it comes about that the Pharmacopœia never entirely covers the range over which the prescribing activity of the medical profession asserts itself. It excludes certain medicines which yet retain a measure of popularity, and it fails to include some of the newer remedies on the ground that no sufficient trial of them has been established. Hence, for these and for others there is no official and authoritative definition, and thus in greater or less degree the prescriber in ordering them is destitute of a secure guarantee that his intentions will be exactly realised. To meet this position various compilations have been prepared by several private firms and individuals, but the very multiplicity of these tends to cause confusion. The issue of this new Codex by the Pharmaceutical Society may be expected to dissipate that confusion, and to establish a uniform standard in the interpretation and dispensing of non-official drugs and preparations when these are ordered in physicians' prescriptions. Hence, *primâ facie*, it would appear to deserve a cordial welcome from the medical profession.

Granted, then, that such a volume is justified, and deferring the question whether or not the Pharmaceutical Society is the appropriate authority to prepare it, attention may be briefly directed to its value to the prescriber. And here common justice at once demands a generous recognition of the care and thoroughness which characterise the whole of the work. There has been no stint of time or ability or money, and many questions which previously had been in an unsettled condition are here brought to a conclusive issue as the result of a specially devised series of researches. It cannot, therefore, be doubted that in its own sphere the book will be recognised as possessing great authority, and, at least so it seems to us, neither prescriber nor dispenser can afford to ignore it. Certainly the former cannot but appreciate the creation, for practically any remedy which he wishes to prescribe, of a standard that commands the adhesion of all pharmacists. Then the very full and carefully prepared index is a ready guide to exact information about almost every remedy and medicinal preparation now before the attention of the profession, and in this respect the book has obviously a large opportunity of service to the medical practitioner. Indeed, it is very likely to become the recognised medium of interpretation between prescribers on the one hand and dispensers on the other. There are also other values which the profession may find in the Codex. Thus very careful and detailed information is given, in connection with each medicine, of the best and most elegant pharmaceutical form in which it can be prescribed, and in this direction pharmacy can give valuable aid to the art of medicine. There are also very useful sections dealing with the extemporaneous preparation of many medicinal applications such, for example, as baths, poultices, enemata, etc. And attention is likewise directed to the combination of remedies and to the risks of incompatible prescriptions. The help that younger practitioners may receive from these cannot but be great, for as we have pointed out elsewhere, one of the most serious defects in the present medical curriculum is the fact that the student receives very meagre instruction in prescribing and dispensing. We would take this opportunity of tendering our thanks to the *Pharmaceutical Journal* for permission to use material from it in the series of articles upon difficulties in "Prescriptions and Dispensing." Thus in numberless points of detail, as well as in its main purpose as an authoritative standard for non-official remedies, the Codex will certainly prove of value to the medical profession. One further point may be mentioned in which it will be serviceable both to medicine and to pharmacy, namely in the relief it will afford from the devices of the private advertiser. In its pages the qualitative and quantitative composition of numerous compound preparations is displayed, and these may be prescribed under appropriate names and quite apart from the commercial interests of particular firms.

A question which will probably excite some controversy is the inclusion in the Codex of sections upon pharmacology and therapeutics. The committee charged with the compilation of the volume has had here the valuable aid of Professor Dixon, and, as was to be expected in these circumstances, what is written about the actions and uses of drugs is of a high order of merit. But it may well be asked whether a non-medical corporation, such as the Pharmaceutical Society, is wise to enter upon the subject of the medicinal use of remedies. The course adopted has obvious dangers and anxieties. But, these apart, we are sure that the Codex offers manifest and considerable advantages, and we believe the profession will not be slow to appreciate its worth.

